# Prevalence and Antimicrobial Resistance of *Enterococcus* Species: A Hospital-Based Study in China

**DOI:** 10.3390/ijerph110303424

**Published:** 2014-03-21

**Authors:** Wei Jia, Gang Li, Wen Wang

**Affiliations:** Medical Experimental Center, General Hospital of Ningxia Medical University, 804 Shengli Street, Yinchuan City 750004, Ningxia Hui Autonomous Region, China; E-Mails: gone.lee@163.com (G.L.); minisat@126.com (W.W.)

**Keywords:** *Enterococcus* spp., antimicrobial resistance, active efflux mechanism, reserpine, fluoroquinolones

## Abstract

*Objective*: to investigate the prevalence and antimicrobial resistance of *Enterococcus* species isolated from a university hospital, and explore the mechanisms underlying the antimicrobial resistance, so as to provide clinical evidence for the inappropriate clinical use of antimicrobial agents and the control and prevention of enterococcal infections. *Methods*: a total of 1,157 enterococcal strains isolated from various clinical specimens from January 2010 to December 2012 in the General Hospital of Ningxia Medical University were identified to species level with a VITEK-2 COMPACT fully automated microbiological system, and the antimicrobial susceptibility of *Enterococcus* species was determined using the Kirby-Bauer disc diffusion method. The multiple-drug resistant enterococcal isolates were screened from the clinical isolates of *Enterococcus* species from the burns department. The minimal inhibitory concentration (MIC) of *Enterococcus* species to the three fluoroquinolones, including ciprofloxacin, gatifloxacin and levofloxacin was determined with the agar dilution method, and the changes in the MIC of *Enterococcus* species to the three fluoroquinolones following reserpine treatment were evaluated. The β-lactam, aminoglycoside, tetracycline, macrolide, glycopeptide resistance genes and the efflux pump *emeA* genes were detected in the enterococcal isolates using a polymerase chain reaction (PCR) assay. *Results*: the 1,157 clinical isolates of *Enterococcus* species included 679 *E. faecium* isolates (58.7%), 382 *E. faecalis* isolates (33%), 26 *E. casseliflavus* isolates (2.2%), 24 *E. avium* isolates (2.1%), and 46 isolates of other *Enterococcus* species (4%). The prevalence of antimicrobial resistance varied significantly between *E. faecium* and *E. faecalis*, and ≤1.1% of these two *Enterococcus* species were found to be resistant to vancomycin, teicoplanin or linezolid. In addition, the *Enterococcus* species isolated from different departments of the hospital exhibited various resistances to the same antimicrobial agent, while reserpine treatment reduced the resistance of *Enterococcus* species to ciprofloxacin, gatifloxacin and levofloxacin. The β-lactamase gene *TEM*, aminoglycoside-modifying-enzyme genes *aac(6')-aph(2")*, *aph(3')-III*, *ant(6)-I* and *ant(2")-I*, tetracycline resistance gene *tetM*, erythromycin resistance gene *ermB*, vancomycin resistance gene *vanA* and the enterococcal multidrug resistance efflux *emeA* gene were detected in 77%, 62%, 26%, 13%, 36%, 31%, 66%, 5% and 55% of the 100 multiple-drug resistant enterococcal isolates. *Conclusions*: similar to previous findings, *E. faecium* and *E. faecalis* are predominant conditionally pathogenic bacteria that cause hospital-acquired infections that can cause urinary and respiratory system infections. Multiple and high-level antimicrobial resistance is highly prevalent in the hospital isolates of *Enterococcus* species. Reserpine treatment inhibits the active efflux of *Enterococcus* species to ciprofloxacin, gatifloxacin and levofloxacin *in vitro* and reduces the MIC of *Enterococcus* species to these three fluoroquinolones. The presence of the enterococcal multidrug resistance efflux *emeA* gene is associated with the resistance to antibiotics in *Enterococcus* species. The monitoring of the prevalence and antimicrobial resistance of *Enterococcus* species is of great significance to guide the control and prevention of enterococcal infections.

## 1. Introduction

Enterococci are commensal bacteria inhabiting the intestines of both humans and animals, which are the major conditionally pathogenic bacteria that cause hospital-acquired infections [1]. Recently, frequent inappropriate use of antimicrobial agents, increase in invasive therapy, and wide use of immunosuppressants has resulted in a growing rise in the number of clinical infections caused by *Enterococcus* spp., notably *Enterococcus faecium* [2]. In addition, the emergence of high-level aminoglycoside-resistant (HLAR) enterococci and vancomycin-resistant enterococci (VRE) causes great difficulties in clinical anti-infective therapy [3,4,5]. In this hospital-based study, a total of 1,157 *Enterococcus* strains isolated from a university hospital during the period from January 2010 through December 2012 were detected and identified to investigate the prevalence and antimicrobial resistance of *Enterococcus* species. In addition the mechanisms underlying the antimicrobial resistance were explored so as to provide clinical evidence for the inappropriate clinical use of antimicrobial agents and the control and prevention of enterococcal infections.

## 2. Materials and Methods

### 2.1. Enterococcus Strains

A total of 1,157 enterococcal strains were isolated from 1,157 diverse clinical specimens obtained from January 2010 to December 2012 at the General Hospital of Ningxia Medical University (Yinchuan, China). All strains were identified to the species level with a VITEK-2 COMPACT fully automated microbiological system (bioMérieux, Inc.; Durham, NC, USA). The quality control strain *Enterococcus faecalis* ATCC 29212 was purchased from Shanghai Harmony Biotechnology Co., Ltd. (Shanghai, China).

### 2.2. Antibiotic Susceptibility Testing

The susceptibility of *Enterococcus* species to 16 antibiotics was determined using the Clinical Laboratory Standard Institute (CLSI) recommended, WHO modified Kirby-Bauer disc diffusion method [6].

### 2.3. Screening of Multiple-drug Resistant Enterococcal Isolates

**Figure 1:**
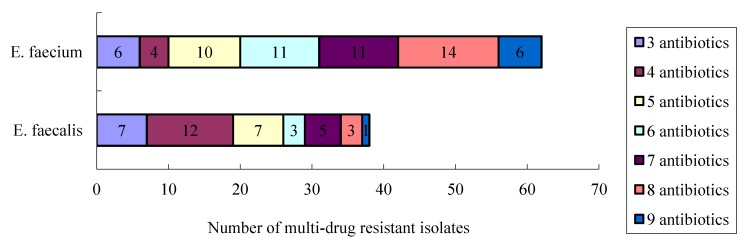
Antimicrobial resistance in 100 multiple-drug resistant enterococcal isolates.

A total of 100 multiple-drug resistant enterococcal isolates (resistant to at least three antibiotics) were screened from the 182 isolates of *Enterococcus* species from the burns department during the period between January 2010 and December 2012, and the antimicrobial resistance in these 100 multiple-drug resistant enterococcal strains is described in Figure 1 and Table 1.

#### 2.3.1. Efflux Pump Inhibition Assay

The minimal inhibitory concentration (MIC) of *Enterococcus* species to the three fluoroquinolones, including ciprofloxacin, gatifloxacin and levofloxacin (Dalian Meilun Biology Technology Co., Ltd.; Dalian, China) at final concentrations of 0.25–512 mg/L, was determined with the agar dilution method [7], while *E. faecalis* ATCC 29212 served as a control isolate. In addition, the MIC of *Enterococcus* species to ciprofloxacin, gatifloxacin and levofloxacin following treatment with an efflux pump inhibitor reserpine (Dalian Meilun Biology Technology Co., Ltd.; (Dalian, China) at a concentration of 20 mg/L, while the antibiotics-free Mueller-Hinton (M-H) agar (Oxoid, Basingstoke, UK) medium containing 20 mg/L reserpine served as controls.

**Table 1:** Antimicrobial resistance in 100 isolates of *E. faecium* and *E. faecalis*.

Antibiotics	*E. faecium* (*n* = 62)		*E. faecalis* (*n* = 38)	
	Antibiotics-resistant isolate	Prevalence (%)	Antibiotics-resistant isolate	Prevalence (%)
Penicillin	55	88.7	2	5.3
Ampicillin	51	82.3	2	5.3
High-level gentamicin	1	1.6	1	2.6
Rifampicin	49	79.0	17	44.7
Ciprofloxacin	36	58.1	6	15.8
Levofloxacin	28	45.2	5	13.2
Fosfomycin	15	24.2	3	7.9
Erythromycin	56	90.3	20	52.6
Furadantin	7	11.3	1	2.6
Linezolid	0	0.0	0	0.0
Vancomycin	0	0.0	0	0.0
Teicoplanin	0	0.0	0	0.0
Chloramphenicol	3	4.8	10	26.3
Quinupristin/dalfopristin	0	0.0	25	65.8
Minocycline	20	32.3	18	47.4
Tetracycline	30	48.4	26	68.4

#### 2.3.2. Detection of Antimicrobial Resistance Genes

The 100 multiple-drug resistant enterococcal strains were isolated in pure cultures. Then, 6–8 enterococcal colonies were randomly selected, diluted with 200 µL of ddH_2_O, centrifuged, boiled at 95 °C for 10 min, followed by centrifugation at 12,000 r/min for 5 min, and the supernatant was the DNA of the enterococcal strains. The β-lactam, aminoglycoside, tetracycline, macrolide, glycopeptide resistance genes and the efflux pump genes were detected in the 100 multiple-drug resistant enterococcal isolates using a polymerase chain reaction (PCR) assay with primers (Table 2) synthesized by the Sangon Biotech (Shanghai) Co., Ltd. (Shanghai, China). PCR was performed with a 25 μL system containing 12.5 μL Premix Taq (BioTeke Biotech Co., Ltd.; Beijing, China), 1 μL DNA template, 1 μL of the forward and reverse primers, and 9.5 μL ddH_2_O under the following conditions: pre-degeneration at 94 °C for 3 min (at 93 °C for 2 min for the *ant(6)-I* and *tetM* genes and at 94 °C for 5 min for the *emeA* gene), followed by 35 cycles of degeneration at 94 °C for 40 s, annealing at 55 °C for 40 s, and extension at 72 °C for 40 s (35 cycles of degeneration at 93 °C for 30 s, annealing at 55 °C for 30 s, and extension at 72 °C for 60 s for the *ant(6)-I* gene; 35 cycles of degeneration at 93 °C for 60 s, annealing at 55 °C for 60 s, and extension at 72 °C for 60 s for the *tetM* gene; 30 cycles of degeneration at 94 °C for 45 s, annealing at 57 °C for 60 s, and extension at 72 °C for 90 s for the *emeA* gene), and final extension at 72 °C for 2 min (for 5 min for the *ant(6)-I* and *tetM* genes and for 10 min for the *emeA* gene). The amplification products were electrophoresed on 1.5% agarose gels (TAKARA Biotechnology (Dalian) Co., Ltd.; Dalian, China). Following electrophoresis, the agarose gels were stained with ethidium bromide for 15 min, and then visualized with a gel imaging analysis system with Quantity One software (Bio Rad; Hercules, CA, USA).

**Table 2:** Sequences of the primers for amplification of antibiotics-resistant genes in *Enterococcus* spp.

Antibiotic-resistant *Enterococcus* spp.	Representative Gene	Sequence (5'-3')	Amplification Product Size (bp)
β-lactam-resistant *Enterococcus* spp.	*TEM*	P1:AGGAAGAGTATGATTCAACA	535
		P2:CTCGTCGTTTGGTATGGC	
Aminoglycoside-resistant *Enterococcus* spp.	*aac(6')/aph(2')*	P1:CCAAGAGCAATAAGGGCATA	220
		P2:CACTATCATAACCACTACCG	
	*aph(3')-II*	P1:GCCGATGTGGATTGCGAAAA	292
		P2:GCTTGATCCCCAGTAAGTCA	
	*ant(6)-I*	P1:ACTGGCTTAATCAATTTGGG	597
		P2:GCCTTTCCGCCACCTCACC	
	*ant(2'')-I*	P1:GAGCGAAATCTGCCGCTCTGG	320
		P2:CTGTTACAACGGACTGGCCGC	
	*ant(4', 4”)*	P1:GCAAGGACCGACAACATTTC	165
		P2:TGGCACAGATGGTCATAACC	
Tetracycline-resistant *Enterococcus* spp.	*tetM*	P1:GTGTGACGAACTTTACCGAA	501
		P2:GCTTTGTATCTCCAAGAACAC	
Macrolide-resistant *Enterococcus* spp.	*ermB*	P1:GAAAAGGTACTAAACCAAATA	616
		P2:AGTAACGGTACTTAAATTGTTTAC	
	*mefA*	P1:ACTATCATTAATCACTAGTGC	346
		P2:TTCTTCTGGTACTAAAAGTGG	
Glycopeptide-resistant *Enterococcus* spp.	*vanA*	P1: GGGAAAACGACAATTGC	732
		P2:GTACAATGCGGCCGTTA	
	*vanB*	P1:CATCGCCGTCCCCGAATTTCAAA	297
		P2:GATGCGGAAGATACCGTGGCT	
	*vanC1*	P1:GGTATCAAGGAAACCTC	822
		P2:CTTCCGCCATCATAGCT	
	*vanC2/3*	P1:CTCCTACGATTCTCTTG	439
		P2:CGAGCAAGACCTTTAAG	
Multidrug resistance efflux pump	*emeA*	P1:GTGACAGCCTTTGTGGCAGAT	687
		P2:TAGTCCGTTGATGGTTCCTTG	

### 2.4. Statistical Analysis

All data were managed using the software WHONET version 5.6, and all statistical analyses were performed with the statistical software SPSS version 17.0 (SPSS Inc.; Chicago, IL, USA). The difference of antimicrobial sensitivity in *Enterococcus* species was compared with chi-square test, with a *p*-value < 0.05 indicative of statistical significance.

## 3. Results

### 3.1. Distribution of Enterococcus Species in Various Clinical Specimens

The 1,157 *Enterococcus* species were isolated from 1,157 clinical specimens collected between January 2010 to December 2012 in the hospital, including 679 *E. faecium* isolates (58.7%, 679/1,157), 382 *E. faecalis* isolates (33%, 382/1,157), 26 *E. casseliflavus* isolates (2.2%, 26/1,157), 24 *E. avium* isolates (2.1%, 24/1,157), and 46 isolates of other *Enterococcus* species (4%, 46/1,157). The MIC_50_ and MIC_90_ values of the 16 antibiotics against the four major enterococcal strains are shown in Table 3. The top five departments from which *Enterococcus* species were isolated (Table 4) included the burns department (15.7%), intensive care unit (ICU; 14.4%), pediatrics department (13.5%), urology department (5.8%) and respiratory medicine department (4.1%), and the highest prevalence of *Enterococcus* species was detected in urine specimens (31.4%), followed by pus specimens (24.4%) and secretion specimens (16%).

### 3.2. Sensitivity of Enterococcus Species to Antibiotics

Of the 1,157 *Enterococcus* isolates, a low prevalence of resistance to linezolid, vancomycin and teicoplanin was detected, while over 40% prevalence of resistance to most antibiotics tested in this study was found. The prevalence of antimicrobial resistance in isolates of *E. faecium*, *E. faecalis*, *E. casseliflavus* and *E. avium* is presented in Table 5.

### 3.3. Comparison of Antimicrobial Resistance between E. faecium and E. faecalis

*E. faecium* and *E. faecalis* comprised 91.7% of the 1,157 *Enterococcus* species isolates collected from the hospital from January 2010 to December 2012. A significantly higher prevalence of resistance to penicillin, ampicillin, rifampicin, ciprofloxacin, levofloxacin, fosfomycin, erythromycin and furadantin was detected in *E. faecium* than that in *E. faecalis* (*p* < 0.05), while a greater prevalence of resistance to chloramphenicol, quinupristin/dalfopristin, minocycline and tetracycline was found in *E. faecalis* than that in *E. faecium* (*p* < 0.05). In addition, a low prevalence of resistance to linezolid, vancomycin and teicoplanin was detected in both *E. faecium* and *E. faecalis*.

### 3.4. Antimicrobial Resistance in Enterococcus Species Isolated from Various Departments of the Hospital

The prevalence of antimicrobial resistance varied in the *Enterococcus* species isolated from different departments of the hospital. A lower prevalence was detected in the *Enterococcus* species isolated from the department of pediatrics, where a high prevalence of penicillin resistance was found, while the highest prevalence was found in the burns department (Table 6).

**Table 3:** MIC_50_ and MIC_90_ scales of 16 antibiotics against *Enterococcus* species (µg/mL).

Antibiotics	*E. faecium* (*n* = 679)		*E. faecalis* (*n* = 382)		*E. casseliflavus* (*n* = 26)	*E. avium* (*n* = 24)		
	MIC_50_	MIC_90_	MIC_50_	MIC_90_	MIC_50_	MIC_90_	MIC_50_	MIC_90_
Penicillin	64	64	2	8	0.5	2	1	64
Ampicillin	32	32	2	16	2	2	2	16
High-level gentamicin *	-	-	-	-	-	-	-	-
Rifampicin	16	16	8	32	1	2	2	2
Ciprofloxacin	64	128	1	16	0.5	2	0.5	1
Levofloxacin	8	128	1	8	2	4	1	2
Fosfomycin	64	128	32	64	64	256	32	32
Erythromycin	64	256	16	256	1	8	8	8
Furadantin	64	256	16	16	16	32	32	128
Linezolid	2	2	2	2	2	4	1	2
Vancomycin	1	1	1	2	2	4	0.5	1
Teicoplanin	2	4	2	2	4	8	2	2
Chloramphenicol	8	16	8	32	2	4	2	4
Quinupristin/dalfopristin	0.5	1	4	8	1	2	2	4
Minocycline	8	32	16	64	2	8	4	8
Tetracycline	8	16	16	16	1	16	16	16

Note: * Only resistance found was against high-level gentamicin.

**Table 4:** Distribution of 1157 *Enterococcus* species isolated from various clinical departments.

Clinical department	*E. faecium* (*n* = 679)	*E. faecalis* (*n* = 382)	*E. casseliflavus* (*n* = 26)	*E. avium (n* = 24)	*E. raffinosus* (*n* = 18)	*E. gallinarum* (*n* = 7)	Other *Enterococcus* species (*n* = 21)
Department of burns	73	93	7	2	2	2	3
ICU	116	37	4	3	1	1	5
Department of pediatrics	99	44	5	1	3	2	2
Department of urology	27	38	0	1	1	0	0
Department of respiratory medicine	42	5	1	0	0	0	0
Department of: hepatobiliary surgery	30	8	5	0	3	0	1
Department of orthopedics	16	18	1	2	0	1	3
Department of endocrinology	8	9	0	1	1	0	1
Department of neurology	13	4	1	0	0	0	0
Department of gynecology	6	8	0	0	2	0	0
Other department	249	118	2	14	5	1	6

**Table 5:** Antimicrobial resistance in *Enterococcus* species.

Antibiotics	*E. faecium* (*n* = 679)		*E. faecalis* (*n* = 382)		*E. casseliflavus* (*n* = 26)		*E. avium* (*n* = 24)	
	Antibiotics-Resistant Isolate	Prevalence (%)	Antibiotics-resistant Isolate	Prevalence (%)	Antibiotics-Resistant Isolate	Prevalence (%)	Antibiotics-Resistant Isolate	Prevalence (%)
Penicillin	621	91.4	22	5.8	1	3.8	8	33.3
Ampicillin	610	89.8	9	2.4	0	0.0	6	25.0
High-level gentamicin	22	3.2	8	2.1	0	0.0	1	4.5
Rifampicin	566	83.3	191	50.0	0	0.0	0	0.0
Ciprofloxacin	585	86.1	66	17.4	1	3.8	1	4.5
Levofloxacin	552	81.3	65	17.1	1	3.8	0	0.0
Fosfomycin	170	25.0	35	9.1	9	33.3	0	0.0
Erythromycin	615	90.6	235	61.5	8	32.0	22	91.7
Furadantin	238	35.0	10	2.6	0	0.0	5	20.0
Linezolid	6	0.9	4	1.1	0	0.0	0	0.0
Vancomycin	5	0.7	0	0.0	0	0.0	0	0.0
Teicoplanin	4	0.6	0	0.0	2	7.1	0	0.0
Chloramphenicol	65	9.5	149	39.1	0	0.0	0	0.0
Quinupristin/dalfopristin	12	1.8	310	81.2	1	3.8	4	17.4
Minocycline	272	40.0	200	52.4	2	7.1	4	17.4
Tetracycline	360	53.0	277	72.5	6	23.1	18	73.9

**Table 6:** Prevalence of antimicrobial resistance in *Enterococcus* species isolated from different departments of the hospital (%).

Antibiotics	Department of burns (*n* = 182), ICU (*n* = 171)				Department of pediatrics (*n* = 164)	
	*E. faecium*	*E. faecalis*	*E. faecium*	*E. faecalis*	*E. faecium*	*E. faecalis*
Penicillin	81.2	4.5	88.0	11.4	93.6	2.5
Ampicillin	77.9	2.2	89.0	2.7	92.2	0.0
Gentamicin	5.5	0.0	4.3	0.0	1.5	0.0
Ciprofloxacin	85.8	8.9	83.2	18.9	77.1	4.7
Levofloxacin	86.2	7.9	84.7	17.1	60.5	2.5
Erythromycin	83.8	47.3	89.9	59.5	92.9	38.6
Furadantin	20.0	3.4	40.7	2.9	10.0	0.0
Quinupristin/dalfopristin	1.5	77.2	0.0	86.5	1.0	66.7
Tetracycline	62.7	67.4	50.0	67.6	70.0	69.0

### 3.5. Effect of Reserpine Treatment on Antimicrobial Sensitivity in Enterococcus Species

All 100 of the clinical isolates of enterococci grew well on the M-H agar plates with or without reserpine, indicating that reserpine had no inhibitory effects on the growth of *Enterococcus* species. The number of enterococcal isolates resistant to ciprofloxacin, gatifloxacin and levofloxacin was reduced from 42 to 30 following treatment with 20 mg/L reserpine, with a corresponding reduction in the prevalence of resistance from 42% to 30%, while the number of enterococcal isolates resistant to the all three fluoroquinolones was reduced from 30 to 15, with a significant reduction also observed. The MIC alteration of three fluoroquinolones for 100 multiple-drug enterococcal strains before and after reserpine treatment is shown in Table 7. Reduced MIC was observed in 84 clinical isolates of *Enterococcus* species following reserpine treatment, including 72 isolates with increased sensitivity to ciprofloxacin, 55 isolates with increased sensitivity to gatifloxacin and 39 isolates with increased sensitivity to levofloxacin. Following reserpine treatment, 36 isolates had an increased sensitivity to all the three fluoroquinolones, 18 isolated showed an increased sensitivity to two fluoroquinolones, while 30 isolates exhibited an increased sensitivity to a fluoroquinolone (Table 8).

### 3.6. Prevalence of Antimicrobial Resistance Genes

Of the 100 multiple-drug resistant enterococcal isolates, there were 38 isolates of *E. faecalis* and 62 isolates of *E. faecium*, while the *TEM*, *aac(6')/aph(2'')*, *aph(3')-III*, *ant(6)-I*, *ant(2'')-I*, *tetM*, *ermB*, *vanA* and *emeA* genes were detected in 77, 62, 26, 13, 36, 31, 66, 5 and 55 multiple-drug resistant enterococcal isolates, respectively. The detection of these antimicrobial resistance genes in 38 isolates of *E. faecalis* and 62 isolates of *E. faecium* is shown in Table 9. The *emeA* gene was detected in 73.8% of the ciprofloxacin-resistant enterococcal isolates, 76.7% of the gatifloxacin-resistant enterococcal isolates, and 75.8% of the levofloxacin-resistant enterococcal isolates, while the prevalence of the *emeA* gene was 41.4%, 45.7% and 44.8% in the ciprofloxacin-, gatifloxacin- and levofloxacin- sensitive enterococcal isolates, respectively (Table 10).

**Table 7:** Changes in MIC50 and MIC90 of three fluoroquinolones for 100 multiple-drug enterococcal strains before and after reserpine treatment.

Time	Ciprofloxacin			Gatifloxacin			Levofloxacin		
	Prevalence of Drug Resistance (%)	MIC_50_ (mg/L)	MIC_90_ (mg/L)	Prevalence of Drug Resistance (%)	MIC_50_ (mg/L)	MIC_90_ (mg/L)	Prevalence of Drug Resistance (%)	MIC_50_ (mg/L)	MIC_90_ (mg/L)
Before reserpine treatment	42.0	2	256	30.0	1	32	33.0	2	64
After reserpine treatment	28.0	0.25	128	17.0	0.5	8	23.0	2	32

**Table 8:** Changes of antimicrobial sensitivity in 100 enterococcal isolates following treatment with 20 mg/L reserpine.

Antibiotics	Drug Sensitivity Test	No. Isolates	No. of enterococcal Isolates with Reduced MIC following Treatment with 20 mg/L Reserpine				
			MIC Reduction by 1/2	MIC Reduction by 1/4	MIC Reduction by 1/8	MIC Reduction by >1/8	No Reduction
Ciprofloxacin	Resistant	42	10	6	3	21	2
	Sensitive	58	4	9	19	0	26
Gatifloxacin	Resistant	30	7	8	1	13	1
	Sensitive	70	16	4	3	3	44
Levofloxacin	Resistant	33	11	8	5	3	6
	Sensitive	67	11	1	0	0	55

**Table 9:** Detection of antibiotic resistance genes in multiple-drug resistant *E. faecalis* and *E. faecium*.

Antibiotic Resistance Gene	*E. faecalis* Isolate (*n* = 38)		*E. faecium* Isolate (*n* = 62)	
	No. of Isolates with Resistance Gene Detected	Prevalence (%)	No. of Isolates with Resistance Gene Detected	Prevalence (%)
*TEM*	18	47.4	59	95.1
*aac(6’)/aph(2”)*	30	78.9	32	52.4
*Aph(3’)-III*	12	31.6	14	23.3
*Ant(6)-I*	4	10.5	9	14.3
*Ant(2”)-I*	11	28.9	25	39.8
*tetM*	12	31.6	19	30.1
*ermB*	27	71.1	39	62.1
*vanA*	0	0.0	5	8.1
*emeA*	10	26.3	45	72.6

**Table 10:** Prevalence of the *emeA* gene in multiple-drug resistant enterococcal isolates.

Antibiotics	Antibiotic-resistant enterococcal Isolate			Antibiotic-sensitive enterococcal Isolate			χ^2^	*p*
	Total Isolates	No. Isolate with *emeA* Gene Detected	Prevalence (%)	Total Isolates	No. Isolate with *emeA* Gene Detected	Prevalence (%)		
Ciprofloxacin	42	31	73.8%	58	24	41.4	13.02	<0.005
Gatifloxacin	30	23	76.7%	70	32	45.7	8.13	<0.005
Levofloxacin	33	25	75.8%	67	30	44.8	8.57	<0.005

## 4. Discussion

Due to the spread of enterococcal antimicrobial resistance [8,9], the tracing of the infectious sources is of great significance for the control of enterococcal infections and its spreading. Among the 289 enterococcal strainss isolated from a tertiary-care pediatric hospital in Mexico City during an 18-month period, *E. faecalis* and *E. faecium* comprised 81.2% of the total isolates, and antimicrobial resistance in *Enterococcus* spp. was found to be common [10]. Of the 415 enterococcal isolates obtained from clinical samples between January 1999 and 31 December 2001 in the Mubarak Al-Kabeer, Amiri, Adan, Ibn Sina and Maternity hospitals in Kuwait, *E. faecalis* (85.3%) and *E. faecium* (7.7%) accounted for 93% of the samples [11]. Salem-Bekhit and colleagues identified 69.2% *E. faecalis* and 11.3% *E. faecium* in 206 enterococcal species obtained from the clinical samples in Riyadh hospitals of King Saud University, Saudi Arabia [12].

Maschieto *et al.* reported that the distribution of *Enterococcus* spp. isolated from the intestinal tracts of patients from a university hospital in Brazil was *E. faecium* (34%), followed by *E. faecalis* (33%), *E. gallinarum* (23.7%), *E. casseliflavus* (5.2%), *E. avium* (1%), and *E. hirae* (1%) [13]. In China, *E. faecium* and *E. faecalis* were also found to be predominant in the enterococci isolated from clinical specimens [14,15,16,17]. Similar to these findings, the current study showed that *E. faecium* (58.7%) and *E. faecalis* (33%) were predominant in the 1157 clinical isolates of *Enterococcus* species isolated from our hospital. However, the present study involved a large sample size, compared the antimicrobial resistance in enterococcal strains isolated from different departments of the hospital, and investigated the efflux mechanism of resistance in enterococci, which is rarely reported previously. The *Enterococcus* species were mainly isolated from the urinary system clinical specimens, which was in agreement with the detection of *Enterococcus* species isolated from the First Affiliated Hospital of Chongqing Medical University [18]. In addition, *Enterococcus* species were found to be predominantly isolated from the burns department, ICU and pediatrics department, which was associated with the patients’ critical illness, long-term antibiotic use and decline in immune function [19].

*Enterococcus* species are found to be intrinsically resistant to cephalosporins and aminoglycosides. Even though bacteria were found to be sensitive to these drugs in *in-vitro* experiments, unsatisfactory efficacy was found in clinical practice [2,20,21]. Multiple-antimicrobial resistance has been widely reported in *Enterococcus* species [22,23,24,25].

In the current study, a significantly higher prevalence of resistance to penicillin, ampicillin, rifampicin, ciprofloxacin, levofloxacin, fosfomycin, erythromycin and furadantin was detected in *E. faecium* than in *E. faecalis* (*p* < 0.05), while a greater prevalence of resistance to chloramphenicol, quinupristin/dalfopristin, minocycline and tetracycline was found in *E. faecalis* than in *E. faecium* (*p* < 0.05). In addition, a low prevalence of resistance to linezolid, vancomycin and teicoplanin was detected in both *E. faecium* and *E. faecalis*. Therefore, linezolid, vancomycin and teicoplanin are currently widely used drugs for the effective treatment of enterococcal infections [22,23,26]. Quinupristin/dalfopristin, a novel streptogramin antibiotic agent, has been widely used for the treatment of vancomycin-resistant enterococcal infections in USA and Europe, and a high therapeutic efficacy has been achieved [27,28,29]. The mechanism of action of the agent is found to involve early and late stage inhibition of bacterial protein synthesis [30,31], however, the drug shows poor efficacy against *E. faecalis* [32,33]. High rates of resistance to quinupristin-dalfopristin have been detected in enterococci isolated from poultry production environments [34], chickens [35], and clinical specimens [36,37,38]. In the present study, the prevalence of quinupristin-dalfopristin resistance was 81.2% in *E. faecalis*, which was significantly higher than that the 1.8% in *E. faecium* (*p* < 0.05). In addition, quinupristin/dalfopristin has been recommended by CLSI for the treatment of vancomycin-resistant enterococcal infections. Since antimicrobial resistance varies in *Enterococcus* species, there is a great need to identify enterococcal strains to the species level, which would facilitate the appropriate selection of antibiotics.

Like previous reports [16,17], our findings also found that the prevalence of antimicrobial resistance varied in the enterococci isolated from different departments of the hospital. A lower prevalence of antibiotic resistance was detected in the enterococci isolated from the department of pediatrics as compared to those from other departments of the hospital, while a high prevalence of penicillin resistance was found, which may be associated with the frequent application of penicillin, a commonly used drug in pediatrics. A high prevalence of antimicrobial resistance was found in the enterococci isolated from the burns department and ICU of the hospital, which may be attributed to the patients’ critical illness, poor immunity and long-term antibiotic use, or the habit of the antibiotic use [19].

To understand the shift of antimicrobial resistance in enterococci in our hospital, we compared the results from this study to the distribution of antimicrobial resistance in enterococci isolated from clinical specimens during the period from January 2007 through December 2009 [39], and found a great rise in the number of enterococcal isolates, in which *E. faecium* was still predominant, but its constituent ratio increased. In addition, the enterococcal isolates were still resistant to more than 40% of the commonly used antibiotics; however, no significant rise was found in the prevalence of antimicrobial resistance. In the current study, we identified 10 linezolid-resistant enterococcal strains, which were not detected in the enterococci isolated between 2007 and 2009. It is considered that the continuous antibiotic pressure causes the secondary resistance to linezolid in enterococci [40].

Reserpine has been proved to reduce the MIC of fluoroquinolones against antimicrobial-resistant bacteria [41,42,43]. It is found that the combination of the multidrug efflux inhibitor reserpine and fluoroquinolone enhances the sensitivity of fluoroquinolone-resistant *Streptococcus pneumonia* and *Staphylococcus aureus* to fluoroquinolones [44]. Our findings showed that reserpine treatment caused a significant reduction in the resistance to the three fluoroquinolones ciprofloxacin, gatifloxacin and levofloxacin in *Enterococcus* species, and the MIC of fluoroquinolones was reduced by over 2-fold in 72% of the enterococcal isolates.

In the current study, the *emeA* gene was detected in 73.8% of the ciprofloxacin-resistant enterococci, 76.7% of the gatifloxacin-resistant enterococci, and 75.8% of the levofloxacin-resistant enterococci, respectively, suggesting the presence of other mechanisms involved in the resistance of enterococci to the three fluoroquinolones in addition to drug efflux, and such a gene was present in 41.4% of the ciprofloxacin-sensitive enterococci, 45.7% of the gatifloxacin-sensitive enterococci, and 44.8% of the levofloxacin-sensitive enterococci, respectively, indicating no expression of the *emeA* gene in some enterococcal isolates. In addition, the occurrence of the *emeA* gene was significantly greater in the fluoroquinolone-resistant enterococci than that in the fluoroquinolone-sensitive enterococci (*p* < 0.05), indicating that the distribution of the *emeA* gene was associated with the resistance to the three fluoroquinolones in the *Enterococcus* species.

It is indicated that the resistance of enterococci to β-lactam is caused by the production of β-lactamase, which is encoded by the *TEM* gene, or modification in the penicillin-binding proteins (PBPs) [45,46]. In the current study, a high prevalence of penicillin resistance was detected in *E. faecium*, while a low prevalence was found in *E. faecalis*, and the occurrence of the *TEM* gene was 95.1% and 47.4% in *E. faecium* and *E. faecalis*, respectively. The aminoglycosides resistance in enterococci is mainly attributable to the production of aminoglycoside-modifying enzymes [47]. Currently, over 30 aminoglycoside modifying enzymes have been identified, in which bifunctional 6'-aminoglycoside acetyltransferase (AAC(6')) 2"-aminoglycoside phosphotransferase (APH(2")) enzyme encoded by the *aac(6')/aph(2")* gene is the most common one, which eliminates the synergistic effect between penicillin or glycopeptide antibiotics and aminoglycosides [48]. Our findings showed that the occurrence of the *aac(6')/aph(2")* gene, the *aph(3')-III* gene that encodes aminoglycoside 3'-type III phosphotransferase (APH(3')-III), the *ant(6)-I* gene that encodes 6-nucleotidyltransferase I (ANT(6)-I) and the *ant(2")-I* gene that encodes aminoglycoside- 2"-O-nucleotidyltransferase I (ANT(2")-I) was 62%, 26%, 13% and 36% in the 100 multiple-drug resistant enterococcal isolates, respectively. The resistance of enterococci to tetracyclines is mainly caused by the binding of the *tetM* gene-encoded ribosomal protection proteins to the ribosome, thereby avoiding the effect of tetracyclines [49]. In the current study, the prevalence of tetracycline resistance gene was 31.6% and 30.1% in *E. faecalis* and *E. faecium*, respectively. It is therefore considered that the resistance of *Enterococcus* species to β-lactam, aminoglycosides and tetracyclines is attributable to the presence of the gene that encodes the corresponding enzymes. Two mechanisms are considered to be responsible for macrolides resistance in enterococci, including the change in the target site of erythromycin mediated by the *erm* gene, and *mef* gene-mediated antimicrobial efflux [50,51]. *ermB* gene is the predominant type of erm gene in enterococci [50]. Our findings showed that the occurrence of the *ermB* gene was 71.1% and 62.1% in *E. faecalis* and *E. faecium*, respectively, indicating that the macrolides resistance in the *Enterococcus* species isolated from Ningxia region is mainly associated with the presence of the *ermB* gene. Like previous studies [52,53], the *mefA* gene was detected in enterococci in the current study; however, Liang *et al.* [54] detected the *mefA* gene in 9 of 53 clinical isolates of *Enterococcus* species, which may be due to the regional variation in the occurrence of the *mefA* gene in enterococci.

It is indicated that the resistance to glycopeptides in enterococci is mainly caused by the alteration of peptidoglycan precursors on the cell wall of enterococci, which leads to the failure of the glycopeptides to inhibiting the synthesis of the cell walls of enterococci, thereby resulting in the emergence of glycopeptide resistance [55,56]. In the present study, the *vanA* gene was detected in all of the 5 vancomycin-resistant isolates of enterococci. Vancomycin-resistant enterococci may transfer the *vanA* gene to *S. aureus*, which leads to the emergence of vancomycin-resistant *S. aureus*, thereby resulting in more difficulty in the clinical treatment of enterococcal infections [5]. Therefore, vancomycin should be used cautiously in the clinical therapy of enterococcal infections, and the management of vancomycin-resistant enterococci should be improved [22,57].

## 5. Conclusions

In summary, enterococci have become the major pathogenic bacteria that cause hospital-acquired infections due to multiple-antimicrobial resistance, and the clinical enterococcal infections predominantly occur in the urinary system. Antimicrobial sensitivity varies in different *Enterococcus* species, and the resistance of enterococci to antimicrobial agents is mainly attributable to the emergence of antimicrobial resistance genes. Reserpine, as an active efflux inhibitor, inhibits the active efflux of *Enterococcus* species, and reduces the MIC of antimicrobial-resistant *Enterococcus* species. The occurrence of the enterococcal multidrug resistance efflux emeA gene is associated with the resistance of enterococci to antimicrobial agents. The monitoring of the prevalence and antimicrobial resistance of *Enterococcus* species would provide a guide for the appropriate selection of antibiotics and prevent the occurrence of more antimicrobial-resistant enterococcal isolates.
